# Supplementary material for: Discovery and application of insertion-deletion (INDEL) polymorphisms for QTL mapping of early life-history traits in Atlantic salmon
Source: BMC Genomics. 2010 Mar 8;11:156. doi: 10.1186/1471-2164-11-156 (PMC2838853; doi:10.1186/1471-2164-11-156)
Supplement: Additional file 2 — Information on developed 76 locus single-run INDEL panel in Atlantic salmon. Information on fluorescence labeling, primer concentrations, PCR pooling and links to alignments, INDEL motifs and GENESCAN (Burge and Karlin 1997) predictions of genes/exons are available in html format. [file 1471-2164-11-156-S2.ZIP › Additionalfile2/snpsummary467.html]

```
Cluster 46 Contig 2

prev  Summary    Contig List  next
```

Size of Consensus sequence = 1161

Number of sequences = 6

Minimum redundancy = 2

Key

A gi|89875255|gb|DY731378.1|DY731378 EST\_ssal\_rgb2\_87117 ssalrgb2 mixed\_tissue Salmo salar cDNA Salmo salar cDNA clone ssal\_rgb2\_642\_212\_rev 5', mRNA sequence  
B gi|89840275|gb|DY698190.1|DY698190 EST\_ssal\_rgb2\_53929 ssalrgb2 mixed\_tissue Salmo salar cDNA Salmo salar cDNA clone ssal\_rgb2\_587\_078\_rev 5', mRNA sequence  
C gi|117433000|gb|EG765224.1|EG765224 EST\_ssal\_sjb\_3022 ssalsjb mixed\_tissue Salmo salar cDNA Salmo salar cDNA clone ssal\_sjb\_009\_157\_fwd 3', mRNA sequence  
D gi|117426327|gb|EG758551.1|EG758551 EST\_ssal\_sjb\_7101 ssalsjb mixed\_tissue Salmo salar cDNA Salmo salar cDNA clone ssal\_sjb\_015\_151\_fwd 3', mRNA sequence  
E gi|117426326|gb|EG758550.1|EG758550 EST\_ssal\_sjb\_7100 ssalsjb mixed\_tissue Salmo salar cDNA Salmo salar cDNA clone ssal\_sjb\_015\_151\_rev 5', mRNA sequence  
F gi|117433001|gb|EG765225.1|EG765225 EST\_ssal\_sjb\_3023 ssalsjb mixed\_tissue Salmo salar cDNA Salmo salar cDNA clone ssal\_sjb\_009\_157\_rev 5', mRNA sequence

13 SNPs detected

A B C D E F  cosegregation weighted

441 C C - C . -   1/13 6.41
504 T T - - - -   11/13 84.62
505 C C - - - -   11/13 84.62
506 A A - - - -   11/13 84.62
507 T T - - - -   11/13 84.62
508 A A - - - -   11/13 84.62
509 G G - - - -   11/13 84.62
510 C C - - - -   11/13 84.62
511 A A - - - -   11/13 84.62
512 G G - - - -   11/13 84.62
513 T T - - - -   11/13 84.62
514 T T - - - -   11/13 84.62
575 C C C T T C   1/13 7.69
